# Spatial-Temporal Pattern of Human-Induced Land Degradation in Northern China in the Past 3 Decades—RESTREND Approach

**DOI:** 10.3390/ijerph16132258

**Published:** 2019-06-26

**Authors:** Wenyi Zhuge, Yaojie Yue, Yanrui Shang

**Affiliations:** 1 Key Laboratory of Environmental Change and Natural Disaster of Ministry of Education; Faculty of Geographical Science, Beijing Normal University, Beijing 100875, China; 2 College of Resources and Environment Science, Hebei Normal University; Key laboratory of environmental evolution and ecological construction of Hebei province, Shijiazhuang 050024, China

**Keywords:** human-induced land degradation, spatial-temporal pattern, RESTREND approach, “optimal cumulative precipitation-NDVImax” regression model, northern China

## Abstract

Land degradation is one of the world’s most serious environmental issues. Human activities play an important role in it. Therefore, human-induced land degradation monitoring is of crucial scientific significance in revealing the evolution of land degradation and guiding its governance. Based on the residual trend (RESTREND) approach and using Global Inventory Modeling and Mapping Studies (GIMMS) normalized difference vegetation index (NDVI) 3g and monthly precipitation as data sources, a quantitative evaluation is conducted on the conditions of human-induced land degradation during 1982–2012 in northern China. The results indicate that (1) the “optimal cumulative precipitation-NDVImax” regression model constructed herein can improve the capability of recognizing human-induced land degradation of arid and semiarid areas in the RESTREND approach. Moreover, long time-series NDVI and precipitation data may reduce the uncertainty of quantifying human-induced land degradation. (2) In the past 3 decades, northern China has experienced three stages of human-induced land degradation, i.e., rapid development, overall reversal with local development, and continuous reversion. Human-induced land degradation in the agro-pastoral ecotone of northern China has shown a rapid restoration trend since the 1990s. (3) It is believed that the dominant factor of land degradation has a significant spatial-temporal scale effect and spatial heterogeneity. Therefore, concrete issues should be specifically analyzed to improve our understanding of land degradation development and reversal, the spatial-temporal pattern and the driving forces of land degradation in the past 3 decades in northern China. Climate change may be the main driving force of land degradation. However, the influence of human activities on the development and reversal of land degradation in small areas and in a short time is more remarkable.

## 1. Introduction

Land degradation has been defined as a reduction in the capacity of the land to provide ecosystem goods, functions and services [1]. It is one of the gravest environmental issues worldwide, not only causing a serious adverse effect on the ecological environment, social economy and politics but also restricting global sustainable development [2,3,4,5]. Moreover, the world is suffering increasingly severe land degradation under the influence of sustained climate change and unreasonable land use [3,6]. Many terrestrial ecosystems are dominated directly by humanity, and no ecosystem on the Earth’s surface is free of pervasive human influence [7]. Numerous studies have found that human activities play a key role in land degradation expansion [8,9,10]. Therefore, it is urgent to strengthen studies on the spatial-temporal pattern monitoring of human-induced land degradation [11]. However, distinguishing land degradation caused by human activities from that caused by climate change in a scientific way has always been a major scientific challenge [12]. Such a study provides a critical scientific basis for promoting land degradation prevention and sustainable land management [13,14].

One of the key methodologies that has been widely used to dynamically monitor land degradation, among a variety of others, is remote sensing (RS). RS can be used for monitoring land degradation on a large regional scale at a low cost [15]. Within RS, there are three main approaches: image interpretation, index evaluation and residual trends (RESTREND) [16,17,18]. However, image interpretation and index evaluation have been proven to be highly subjective, laborious and time- consuming when applied on a large scale [3,19]. More importantly, whether in index evaluation or image interpretation, one of the challenges is separating human from climate signals, which is why RESTREND was developed.

The principle of RESTREND is to use the normalized difference vegetation index (NDVI) residual (the differences between observed and predicted NDVI values) trend to reflect land degradation under the influence of human activities [20]. RESTREND was initially used for the monitoring of human-induced land degradation in Syria and was thereafter applied and improved by Wessels et al., Li et al., and Burrell et al. [21,22,23,24]. The basic assumption of RESTREND is that there is a strong correlation between vegetation production and precipitation in water-restricted ecosystems [25]. Since vegetation change is generally deemed an important indicator of degradation, this method is widely applied in land degradation arising from human activities. However, such an approach also has disadvantages. For instance, the residual trend is affected by the selected time series, and not all negative trends indicate land degradation [21,22,26]. Therefore, when using RESTREND for land degradation monitoring, it is necessary to analyze the specific conditions in the area in which there is a negative trend and to select the appropriate starting and ending locations of the time series [21].

China is one of the countries with the most serious land degradation in the world [3]. There are 13 deserts and sandy lands and a large amount of desertification land in northern China [27]. Furthermore, the combination of climate change and irrational human activities has led to a high risk of land degradation in northern China [27]. Some scholars believe that China’s land degradation is mainly caused by human activities and that climate change plays a positive role [8,28]. However, the long-term spatial and temporal patterns of human-induced land degradation in northern China have rarely been studied.

Therefore, using northern China as an example, the main goal of this study is to use the RESTREND method for quantifying the spatial-temporal pattern of human-induced land degradation. In the following sections, we will first describe the methodological framework of the RESTREND method. Then, the spatial-temporal pattern of precipitation and human-induced land degradation in northern China will be quantitatively shown. Finally, we will analyze the applicability of the RESTREND method in northern China and further discuss the regional differentiation and the spatiotemporal scale effect of the dominant factors for land degradation.

## 2. Approaches and Data Sources

### 2.1. Overview of Study Areas

**Figure 1:**
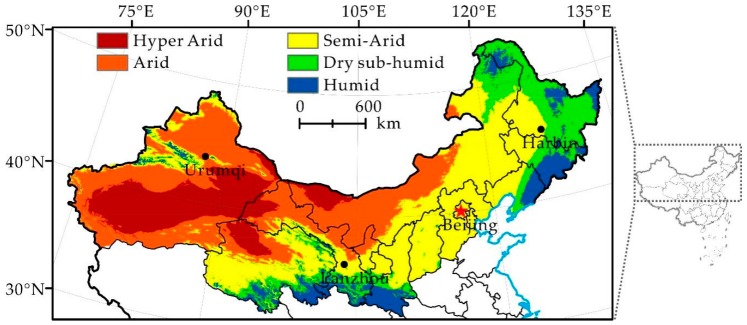
Location of the Study Areas [32].

The study areas (73° E–136° E, 31° N–54° N, Figure 1) consist of 13 provinces, municipalities and autonomous regions of northern China, including Heilongjiang, Jilin, Liaoning, Inner Mongolia, Beijing, Tianjin, Hebei, Shanxi, Shaanxi, Ningxia, Gansu, Qinghai and Xinjiang. The total area is approximately 5.3 × 10^6^ km^2^, accounting for approximately 56% of the national land area. The population is 3.8 × 10^8^ (in 2016), accounting for approximately 27% of the national total population.

Most areas of northern China are dry lands [29]. The annual precipitation transitions from 427.5 mm in the southeast to 11.7 mm in the northwest portion of northern China [30]. An area of 4.13 × 10^6^ km^2^ is distributed in hyper-arid, arid, and semi-arid regions, accounting for 77.98% of the total northern China land area (Figure 1). Therefore, precipitation has a significant impact on vegetation coverage in this region [30]. With less precipitation and strong wind forces, the ecological environment in northern China is fragile and is susceptible to the occurrence of land degradation due to human impact [29]. For example, it is estimated that 90% of China’s grasslands, which are mainly distributed in northern China, are now considered degraded [31]. Hence, the fragile ecosystems in the study area are prone to land degradation [10].

### 2.2. Detection Approach of Human-Induced Land Degradation

The RESTREND method was adopted for the detection of human-induced land degradation. This approach separates the precipitation/climate signal in NDVI and assumes that the residual signal is caused by human activities [20,21]. The detection of land degradation caused by human activities in northern China includes four steps as follows:

Step 1: Determine the quantitative relationship between the maximum NDVI (NDVImax) in the growing season and the optimal cumulative precipitation in the research period.

The annually accumulated precipitation of seven time scales (January–December, March–July, March–August, April–July, April–August, April–October, June–August) was calculated, and linear regression analyses were performed with the corresponding NDVImax to determine the most suitable linear regression model of NDVImax and optimal cumulative precipitation [23,24,25]. The specific operation method is performed by building random points with ArcGIS in the range of the research areas, then performing regression analyses of the NDVImax of each random point and the corresponding accumulated precipitation from the seven time scales. The optimal cumulative precipitation of every year is selected according to the principle of optimal R^2^ and Sig < 0.001, consequently obtaining the “optimal cumulative precipitation-NDVImax” optimal linear regression model.

Step 2: Calculate the residual trend. First, the potential maximum annual NDVI (NDVIpot) of every pixel was calculated based on the “optimal cumulative precipitation-NDVImax” optimal linear regression model. Second, the NDVIpot is subtracted from the observed NDVImax value. The difference is the residual trend.

Step 3: Analyze the residual trend. The interannual change trend of the pixel-by-pixel fitting of the residual for the unary linear regression trend model based on the least squares method is adopted to obtain its slope, and the residual change trend is reflected through the positive trend, negative trend and size of the slope. Any residual trend is not related to precipitation, which is an indicator representing land degradation [21]. The residual trend may change with the length of different research periods, and the result is uncertain; however, the difference of the result contributes to analyzing the effect of vegetation change and human activities in the different periods [26].

Step 4: Compare land degradation by grading and charting. Fixed starting years are used in this study, and three research periods, i.e., 1982–1990 (short period), 1982–2000 (medium period) and 1982–2012 (long period) are selected as mutual referents. The residual trend of the three periods is analyzed and compared, thereby obtaining the condition of human-induced land degradation in northern China in the 1980s, 1990s and in the 21st century.

The residual change trend was divided into eight land degradation grades based on the residual slope value of the unary linear regression trend line [33] (Table 1). The unary linear regression slope of residuals that D1, D2, D3, and D4 represent was less than 0, and its residual showed a downward trend, manifested as land degradation along with its extent, D1 < D2 < D3 < D4; the unary linear regression slope of residuals that I1, I2, I3 and I4 represent was greater than 0, and its residual showed an upward trend, manifested as land restoration along with its extent, I1 < I2 < I3 < I4. Based on these grades, the pattern and dynamic condition of human-induced land degradation in northern China in the past three decades can be analyzed.

### 2.3. Data Source

The global vegetation data set GIMMS NDVI 3g (downloaded from the ECOCAST website, http://ecocast.arc.nasa.gov) was applied. Its spatial resolution is 8 km × 8 km, and its temporal resolution is 15 days. Although there are many higher spatial resolution NDVI products, such as SPOT (1 km), MERIS (1 km) and MODIS (500 m), GIMMS NDVI 3g continues to be the most popular record due to the unparalleled time span [20]. It is frequently used for the evaluation of long-term change in the vegetation productivity of global and regional land [34]. In the present study, the time series of GIMMS NDVI 3g was July 1981–December 2013, with a duration of 33 years, of which the NDVImax of the data from the growing seasons (April–October) during 1982–2012 were selected as data for analysis.

The precipitation data of 2472 ground stations were obtained from the China meteorological data sharing service network (http://data.cma.cn/). Spatial interpolation was made by means of a thin disk spline method of ANUSPLIN software, thereby generating a 0.5° × 0.5° lattice data set of monthly ground precipitation in China during 1982–2012.

## 3. Results

### 3.1. Precipitation Change Trend of Northern China in the Past 3 Decades

**Figure 2:**
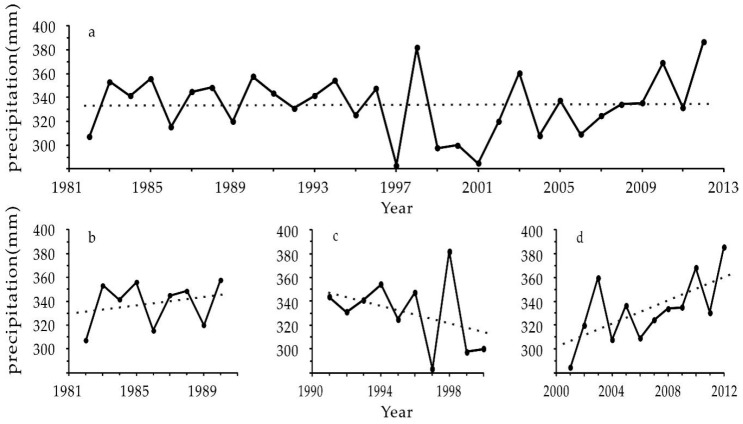
Precipitation Change Trend of Northern China (**a**. 1982–2012; **b**. 1982–1990; **c**. 1991–2000; **d**. 2001–2012).

During 1982–2012, the average annual precipitation of northern China was 333.89 mm, with a slight increase of 0.03 mm per year (Figure 2a). Precipitation shows differences at different stages and in various areas. During 1982–1990, the annual precipitation showed a slow upward trend, at a rate of 1.75 mm per year (Figure 2b). However, the annual precipitation presented a sharp downward trend during 1991–2000, at a rate of 3.72 mm per year, of which the precipitation in 1997 was the lowest in the past three decades (Figure 2c). Then, the annual precipitation showed a faster upward trend after 2000, at a rate of 4.96 mm per year. The annual precipitation in 2012 was 386.46 mm, the highest value in the past 3 decades (Figure 2d).

**Figure 3:**
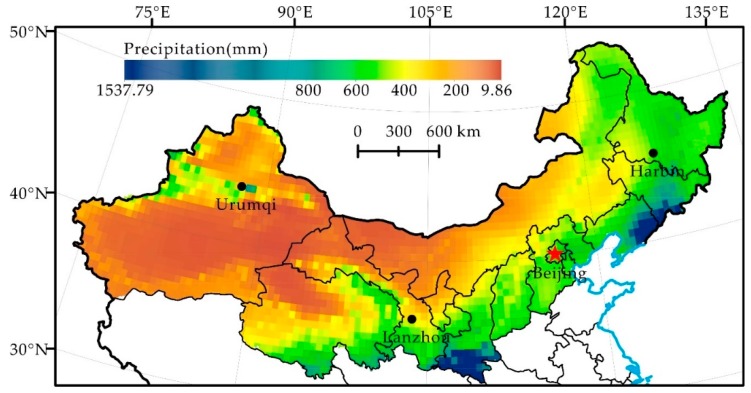
Spatial Distribution of Average Annual Precipitation in Northern China.

The precipitation in northern China had a significant regional difference. Generally, the spatial distribution of annual precipitation showed a decreasing pattern from southeast to northwest (Figure 3). The average annual precipitation in the Northeast, North China, and Northwest areas was 606.97 mm, 481.54 mm, and 359.84 mm, respectively. The areas with high precipitation were mainly located in the Southern Shaanxi, Liaodong Peninsula and Southern Jilin, and those with low precipitation were mainly situated in Western Inner Mongolia, Gansu, Northern Qinghai and Xinjiang, with an average annual precipitation of below 100 mm. The spatial distribution pattern of precipitation in northern China was consistent with that of dry land (Figure 1).

### 3.2. Estimated Result of NDVIpot and Detection in Northern China

**Figure A1:**
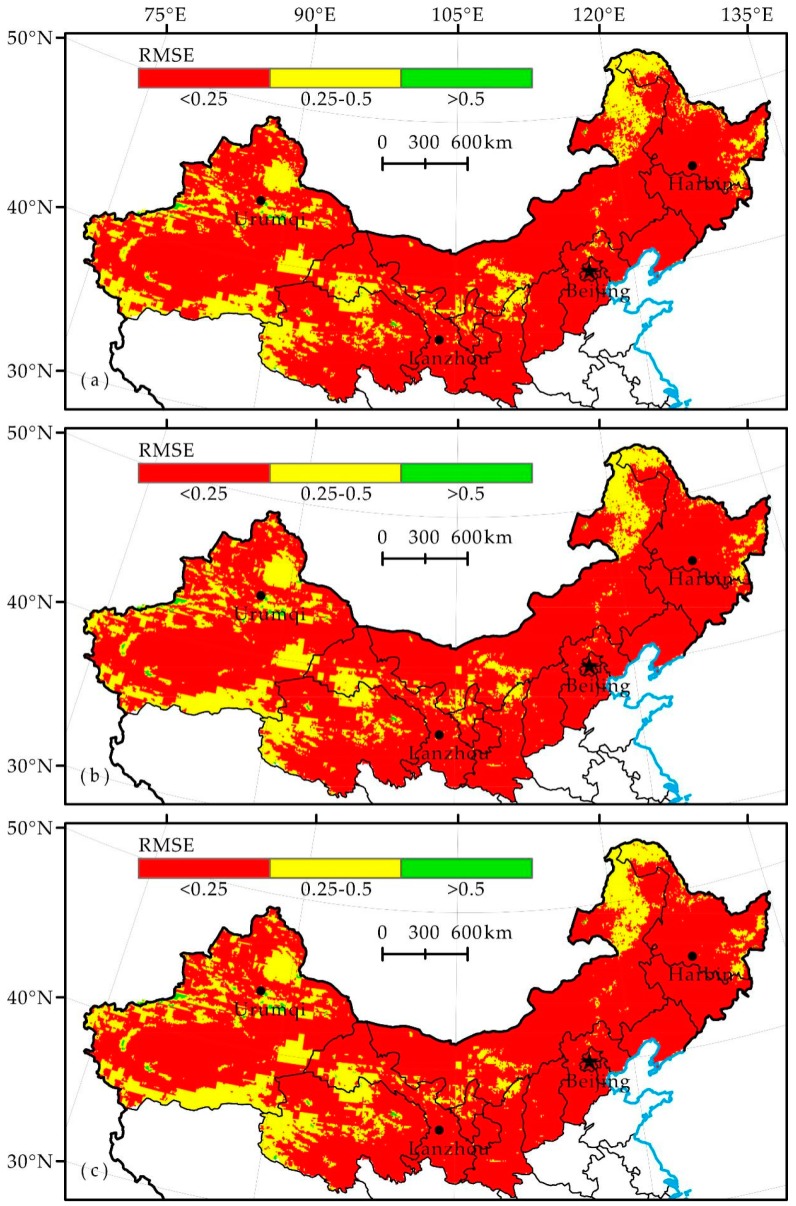
Root Mean Square Error (RMSE) Distribution of NDVIpot and Actual NDVI of Northern China Simulated Based on the Most Suitable Linear Regression Model ((**a**) 1982–1990; (**b**) 1982–2000; (**c**) 1982–2012)).

Table 2 shows the most suitable linear regression model between NDVImax and optimal cumulative precipitation in each year. The result shows that NDVImax had a statistically significant relationship with the optimal cumulative precipitation of every year. According to the comparison between the observed NDVI of northern China and the simulated NDVIpot based on the most suitable linear regression model, the root mean square error (RMSE) was lower overall (Appendix A Figure A1, Table A1). The quantity of pixels less than 0.25 of short-term, medium-term and long-term RMSE was the highest, accounting for 81.5%, 81.4% and 80.8% of all pixels, respectively. The pixels more than 0.5 of RMSE were sporadically distributed in Xinjiang and accounted for no more than 0.4%, indicating that the prediction model of NDVImax constructed based on the optimal cumulative precipitation of every year had high precision, suitable for the prediction of NDVIpot in northern China.

### 3.3. Human-Induced Land Degradation Pattern of Northern China in the Past Three Decades

**Figure 4:**
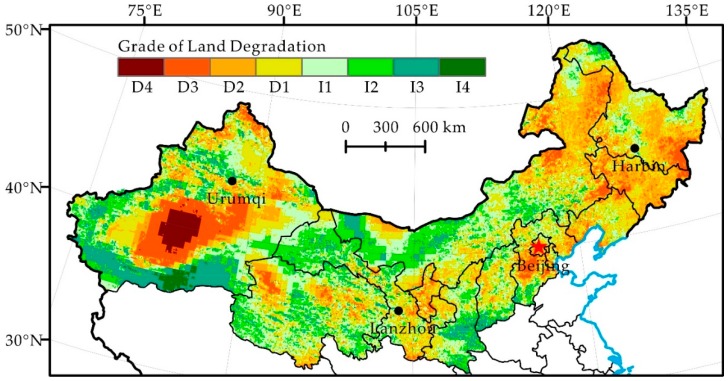
Distribution of Grade of Land Degradation during 1982–1990 in Northern China.

**Figure 5:**
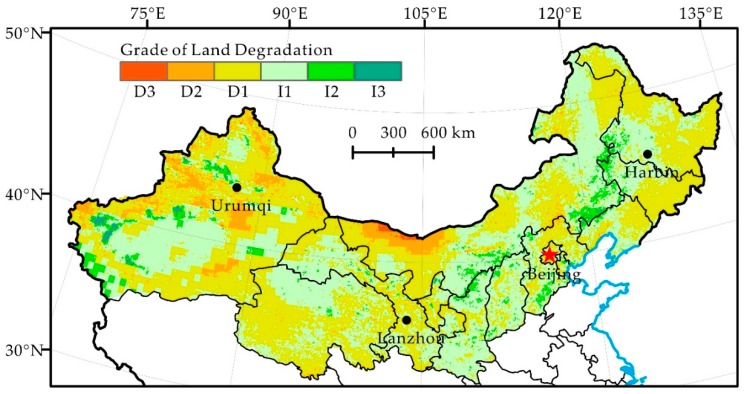
Distribution of Grade of Land Degradation during 1982–2000 in Northern China.

**Figure 6:**
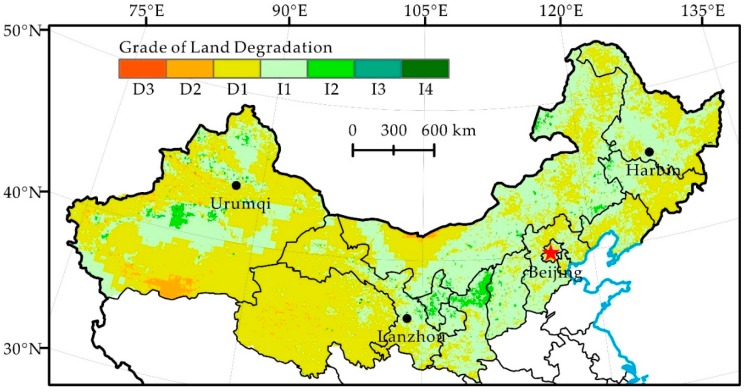
Distribution of Grade of Land Degradation during 1982–2012 in Northern China.

The spatial pattern of short-term, medium-term and long-term human-induced land degradation in northern China during 1982–2012 was determined (Figure 4, Figure 5 and Figure 6). The result shows that the degraded and restored land in northern China presents a staggered distribution pattern in the past three decades, and these areas are characterized by a centralized continuous distribution changing to a staggered distribution.

In the short period (1982–1990) of the past three decades, the human-induced land degradation of northern China shows integral development and local reversal (Figure 4, Table 3). The degree of land degradation was dominated by slight degradation (25.6%), moderate degradation (17.1%) and severe degradation (8.5%), accounting for 51.2% of the total research area. Land degradation was mainly distributed in the Northeast (Heilongjiang, Jilin, Liaoning and Eastern Inner Mongolia) and Ningxia, Ordos region of Inner Mongolia, Southern Gansu, Qinghai and Xinjiang in Central North China and in the Northwest. However, land degradation reversal centers on slight restoration, moderate restoration, and significant restoration, accounting for 46.8% of the total research area, was mainly distributed in a strip including the Tianshan and Kunlun Mountains, Western Gansu and Western Inner Mongolia.

In the medium period (1982–2000) of the past three decades, the land in northern China was dominated by slight degradation and slight restoration, accounting for 46.8% and 42.9% of the total research area, respectively, in a staggered distribution (Figure 5, Table 3). The areas of moderate degradation and severe degradation decreased substantially, mainly distributed in the Northern Alxa Plateau and Xinjiang; the areas of moderate restoration and significant restoration were principally distributed in the junction of Inner Mongolia and Heilongjiang, Jilin and Liaoning, the junction of Inner Mongolia and Shanxi and Shaanxi as well as in the edge of Tarim Basin, Xinjiang. Compared with the period of 1982–1990, the degree of land degradation in the Northwest was lower, and that of North China and the Northeast greatly improved. The land degradation of northern China was under control and developed toward mitigation. The effects of land restoration in some areas were significant.

Seen from the long period (1982–2012) of the past three decades, the land in northern China was dominated by slight degradation and slight restoration, accounting for 51.66% and 44.77% of the total research area, respectively (Figure 6, Table 3). The areas of slight degradation were mainly distributed in Qinghai, Xinjiang in the Northwest and west of Gansu, Alxa Plateau in Inner Mongolia and in the Northeast. The areas of slight restoration were mainly distributed in North China, Ningxia in the Northwest, Northern Shaanxi, the central and eastern part of Inner Mongolia and the Northeast. The areas of above moderate degradation were sporadically distributed in the Xinjiang and Alxa Plateau. The areas of moderate restoration mainly centered on Ningxia, the Mu Us Desert and Xinjiang. Compared with the period of 1982–2000, land degradation was obviously reversed in northern China in the 21st century.

It shows that from the 1980s to the 1990s and into the 21st century, northern China has experienced the process of human-induced land degradation from rapid development to local development with an overall reversal of degradation and then a continuous restoration (Table 3). Although land degradation is still a major ecological issue facing northern China, its intensity is weakening and some degraded land is reversing. Since the 1990s, human-induced land degradation in the agro-pastoral ecotone of northern China has experienced a rapid restoration, particularly in the Songnen, Horqin, Hunshandak, Mu Us Desert, and the adjoining steppe zone, and human-induced land degradation has been significantly restored.

## 4. Discussion

### 4.1. Relationship between NDVI Residual and Land Degradation

The RESTREND method proposed NDVI residuals as an indicator to quantify human-induced land degradation in water-restricted ecosystems [20,25]. The negative trend of residuals is regarded as a signal of human-induced land degradation [21]. Although humid regions account for 8.26% of the study area, most parts of northern China are hyper-arid, arid, semi-arid and dry sub-humid regions (Figure 1). Thus, in northern China, precipitation was significantly positively related to NDVI change [2]. Serious land degradation ultimately resulted in a long-lasting and observable loss of vegetation cover and biomass productivity over time and in space [25]. Thus, the RESTREND method is applicable to northern China.

However, the reduction in residuals did not have a one-to-one correspondence with land degradation. For instance, some positive residual trends may have indicated harmful land-use development, such as expanding cultivation into dry lands [21]. Therefore, not all reductions in residuals indicated degradation [22,26]. Since the relationship between residual reduction and human-induced land degradation is complex, it is necessary to check carefully to determine the cause of the land degradation trends [21].

### 4.2. Scale Effects in Terms of NDVI Pixel Size

Despite the diversity of NDVI data sources, GIMMS NDVI 3g is the only global vegetation dataset that covers the study period [20]. Therefore, the spatial resolution in this study was determined primarily by the GIMMS NDVI 3g dataset. However, northern China includes diverse vegetation types distributed along temperature and precipitation gradients [30]. Furthermore, some desert and desertified ecosystems in northern China may contain sparse vegetation [10]. In addition, in deserts or heavily degraded areas, it is still possible to have patches of dense vegetation. Consequently, the NDVI that we used in this study may not show any vegetation signal for dense vegetation patches of 2 km × 2 km surrounded by bare sand in its coarse resolution. In other words, the low-resolution NDVI may not be able to accurately reveal the actual distribution characteristics of vegetation and may lead to errors in the results. It should also be noted that although using finer-resolution data may improve the accuracy of the analysis and provide more explanatory power, it may also result in noise overwhelming signals [23]. Therefore, future studies are needed to explore the ‘‘optical’’ pixel or patch size in the application of the RESTREND analysis [23].

### 4.3. Dynamics of Human-Induced Land Degradation in Northern China

Human activities and climate change are the two dominant factors in land degradation. From the viewpoint of sustainable land use, it is practical to alleviate or govern land degradation via the adjustment of human activities. However, distinguishing the human impacts on land degradation from those of climate change has traditionally been difficult [9]. RESTREND is a feasible approach to detect human-induced land degradation [20,21,22,23,24,25]. Evans and Geerken verify the applicability of this approach and ascertain its evaluation capability for land degradation [20]. RESTREND is based on the scientific assumption of the strong correlation between vegetation productivity and precipitation [25]. Because precipitation is a dominant factor in vegetation growth in arid and semiarid areas, this approach is applicable to research on land degradation in northern China [35,36,37]. The prediction model of NDVImax based on optimal cumulative precipitation is constructed (Table 2) to ensure the precision of human-induced land degradation detected by the RESTREND approach.

The results showed that human-induced land degradation in northern China in the past three decades presented a reversing trend, but there are still differences in different stages and regions. In space, degraded and restored land are unevenly spread, and the regional difference is significant. The areas with a high degree of degradation are mainly distributed in the northwest inland, and human-induced land degradation in the agro-pastoral ecotone of northern China presents a rapid restoration state, which is consistent with the results of Wang et al. [38]. Although land degradation has been widely considered one of the most serious environmental issues in China, its distribution and cause is still debated [8]. For instance, Yang [39] believes that degraded land under the influence of humans is mainly distributed in Inner Mongolia and in the semiarid agriculture and pasture interlaced zone along the Great Wall. Zhang et al. [40] emphasized that extremely severe degradation areas are mainly distributed in the arid and semiarid areas in North China and the Northwest.

The land degradation of northern China is affected by both natural and human-induced factors, but the dominant factors of different spatial-temporal scales vary [40]. It is believed that different results may be due to multiple scale effects in land degradation and the complexity and uncertainty of human activities in northern China [41]. For example, there are many areas with high land degradation values in the Northwest (Figure 4), where precipitation increases (Table 2) and drought mitigates during 1982–1990. This indicates that climate change itself cannot explain the condition of land degradation [40,42,43]. Therefore, human activities, such as deforestation and overgrazing, are most likely the dominant factors in land degradation development in these areas and in the western parts of the agro-pastoral ecotone of northern China [43]. In the northeast after the 1990s, precipitation decreases, potential evapotranspiration increases and drought intensifies the effects [42] (Figure 2). In contrast to the rapid land degradation reported by Ma et al. [43], we found that the land degradation of the area caused by humans reverses significantly (Figure 5). This signifies that human activities have a decisive effect on the reversal of land degradation, even if climate factors are adverse to the restoration of land degradation.

Clearly, research on the difference in the spatial-temporal scale will result in discrepancies in land degradation detection results. Many research results show that climate change is the dominant factor in land degradation formation and development in northern China, while human activities impact contemporary land degradation [10,18,44]. Land degradation is a long process of gradual change. Therefore, the evaluation of results on the long-time scale can weaken the uncertainty of the results evaluated on the shorter time scale [40]. In summary, it is considered, based on the results of the past three decades, that human-induced land degradation in northern China persists, but the development trend has been reduced since the 1990s. Although the trends of climate change in the different periods and areas are different, human activities are progressing toward the acceleration of land degradation reversal.

### 4.4. Regional Differentiation of Human Factors in Land Degradation of Northern China

**Figure 7:**
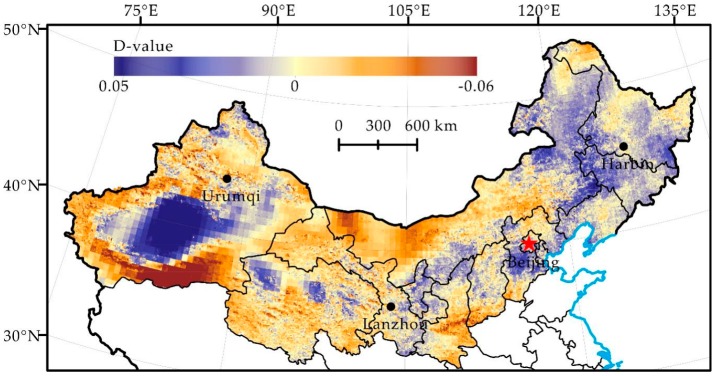
Distribution of Residual Trend Change from 1991 to 2000 (Where D-value positively far away from 0 is a hot spot for land degradation reversal, vice versa, land degradation development).

**Figure 8:**
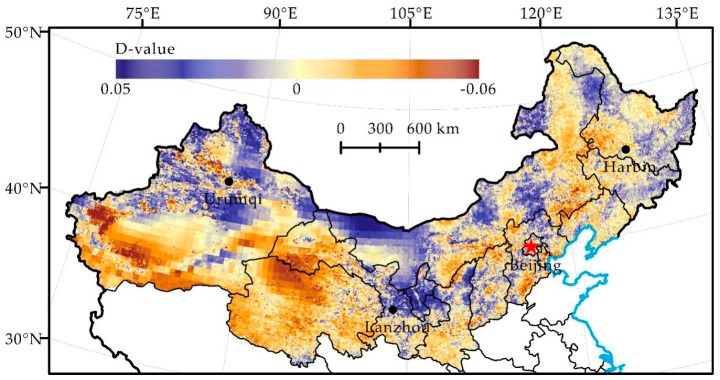
Distribution of Residual Trend Change from 2001 to 2012 (Where D-value positively far away from 0 is a hot spot for land degradation reversal, vice versa, land degradation development).

Our results show that desertification and the relative role of natural and human factors showed obvious spatial heterogeneity, similar to Wei et al. [9]. Therefore, we selected hot spots of land degradation and restoration in northern China (Figure 7 and Figure 8) to further discuss the regional differentiation of the driving force of land degradation and to identify the following three types. 

In the first type, climate change is beneficial to the reversal of land degradation, but the land degradation situation becomes worse, and human activities dominate land degradation. For instance, the Tarim River Basin is affected by increased precipitation and basin flow in the 1980s, and this climate factor is beneficial to the reversal of land degradation [45]. However, our results showed that human-induced land degradation in the area is serious. Our result is consistent with that of Tao et al. [46], Xu et al. [45] and Jiang et al. [47], who also think that human activities such as large-scale land reclamation and excessive water consumption, not climate change, are responsible for the drying and land degradation of the downstream river channels of the Tarim River.

In the second type, human activities play a key role in the development and reversal of land degradation. Our results show that human-induced land degradation of the Alxa Plateau presented a development trend and then gradually reversed after 2000. The climate in this area is more stable and has little effect on land degradation [48]. It reported that land degradation developed under the influence of increased population and livestock in the 1990s [49]. However, an obvious reversal trend since 2000 under the influence of active human activities, such as the reduction of the intensity of agricultural activities and the execution of effective ecological rehabilitation projects, was observed [49]. Therefore, we argue that the development and reversal of land degradation in the Alxa Plateau may be dominated by human activities. It is also pointed out in research by Wang et al. [48] that land degradation of the Alxa Plateau that occurred before 2000 is the consequence of a rapidly increasing population and that the reversal of land degradation that occurred after 2000 is the result of integrated water management implementation.

In the third type, the climate factor contributes to the development of land degradation, but it reverses under the influence of human activities. For instance, the precipitation in the western side of the Northeast China Plain decreased significantly in the 1990s, and the climate factor clearly induced an increase in land degradation [50]. However, we found that land degradation caused by humans reversed significantly, which is similar to the conclusion obtained by Qiu et al. [50], who point out that human factors, such as land protection policy and returning farmland to forests or grassland, dominate the reversal of land degradation in the western side of the Northeast China Plain, instead of detrimental climate change.

Human-induced land degradation in northern China developed rapidly in the 1980s, locally reversed in the 1990s and continued restoration since 2000 (Figure 4, Figure 5 and Figure 6). Its change trend was not the same as the precipitation change in the same period (Figure 2). Analysis of the hot spots of human-induced land degradation indicates that the effect of climate and human factors on land degradation in different areas was complex. However, active human activities, e.g., the Three-North Shelterbelt Project (1978–present), Beijing and Tianjin Sandstorm Source Treatment Project (2001–2010), Returning Farmlands to Forest Project (2003–present), and Returning Grazing Land to Grassland Project (2003–present), are obviously the important driving forces of the reversal of land degradation in northern China [51]. For key fortification objects, the effect of human activities on the mitigation of land degradation is particularly significant [52]. For instance, the land degradation of areas such as Mu Us and Horqin Sandy Lands reversed significantly, fitting the reversal trend of land degradation under the influence of policies such as grazing prohibition [16,53]. In summary, we think that the dominant factor of land degradation and restoration of hot spots is human activities rather than climate change. Climate change may be the long-term driving force of land degradation, but the effect of human activities on the development and reversal of land degradation in the short term is more significant.

Human activities are the most active and primary factor in land use, and artificial disturbance and governance can aggravate or effectively mitigate regional land degradation. Of course, the main driving factor of land degradation is regional disparity [2]. Therefore, land use policies should be formulated for land degradation in northern China according to circumstances [17,54]. Meanwhile, it is necessary to comprehensively consider the unique combination of regional constraints and resources and to take advantage of neglected resources, improving the livelihoods of local residents and promoting environmental conservation [54].

## 5. Conclusions

The RESTREND approach, along with long time-series NDVI and precipitation data from 1982–2012, is adopted herein to detect the spatial-temporal pattern of human-induced land degradation over a short period (1982–1990), a medium period (1982–2000) and a long period (1982–2012) in the past three decades in northern China. In the condition of a random sample, it is found that our proposed model of “optimal cumulative precipitation–NDVImax” consequently improved the prediction ability of the pixel scale NDVI of the RESTREND approach, further enhancing the detection reliability of human-induced land degradation.

During 1982–2012, human-induced degraded land in northern China accounted for approximately 52.2%–53.05% of the total research area. In the past three decades, the degraded land area has not decreased significantly, but the degree of land degradation has obviously decreased. Human-induced land degradation experiences three stages: Rapid development in the 1980s, overall reversal with local development in the 1990s and continuous reversal at the beginning of the 21st century. The areas with severe land degradation are mainly distributed in the Northwest and have had extremely significant restoration, mainly in North China and the Northeast, in particular, the agro-pastoral ecotone. Although the vegetation in northern China has a sensitive response to precipitation change, the precipitation of northern China in the past three decades does not significantly decline. Therefore, the reversal trend of human-induced land degradation presented in the past three decades in northern China may be related to active land protection policies and ecological construction efforts.

The degraded and restored land in northern China is scattered, and the regional disparity is significant, which may be associated with the spatial-temporal scale and spatial heterogeneity of the natural and human factors. On the basis of dynamic analysis of human-induced land degradation in different regions and periods of northern China, we argue that human activities may play an important role in small areas and in short periods.

## Figures and Tables

**Table 1:** Residual Change Trend Grade.

No.	Unary Linear Regression Slope of Residual	Land Degradation Degree
D4	−0.06~−0.03	Extremely severe degradation
D3	−0.03~−0.01	Severe degradation
D2	−0.01~−0.005	Moderate degradation
D1	−0.005~0	Slight degradation
I1	0~0.005	Slight restoration
I2	0.005~0.01	Moderate restoration
I3	0.01~0.03	Significant restoration
I4	0.03~0.06	Extremely significant restoration

**Table 2:** Linear Regression Relationship between Normalized Difference Vegetation Index (NDVI) max and Optimal Cumulative Precipitation during 1982–2012 in Northern China.

Years	x (Optimal Cumulative Precipitation)	R^2^	a	b	F	Sig.
1982	Ln (January–December)	0.628	0.250	−0.882	3336.877	<0.001
1983	Ln (January–December)	0.615	0.228	−0.778	3154.693	<0.001
1984	Ln (April–October)	0.672	0.244	−0.842	4053.405	<0.001
1985	Ln (January–December)	0.588	0.196	−0.595	2821.242	<0.001
1986	Ln (January–December)	0.594	0.216	−0.680	2891.950	<0.001
1987	Ln (January–December)	0.693	0.275	−1.039	4463.948	<0.001
1988	Ln (April–July)	0.692	0.302	−1.008	4454.531	<0.001
1989	Ln (March–July)	0.590	0.233	−0.661	2842.146	<0.001
1990	Ln (January–December)	0.695	0.266	−0.977	4499.123	<0.001
1991	Ln (January–December)	0.633	0.246	−0.869	3409.689	<0.001
1992	Ln (January–December)	0.614	0.264	−0.977	3149.731	<0.001
1993	Ln (January–December)	0.694	0.280	−1.061	4498.016	<0.001
1994	Ln (January–December)	0.610	0.223	−0.731	3095.045	<0.001
1995	Ln (March–July)	0.694	0.242	−0.659	4483.983	<0.001
1996	Ln (January–December)	0.578	0.249	−0.893	2705.651	<0.001
1997	Ln (January–December)	0.617	0.247	−0.852	3187.335	<0.001
1998	Ln (January–December)	0.675	0.262	−0.984	4118.550	<0.001
1999	Ln (January–December)	0.567	0.250	−0.851	2588.770	<0.001
2000	Ln (January–December)	0.610	0.249	−0.858	3091.149	<0.001
2001	Ln (March–July)	0.627	0.214	−0.513	3332.700	<0.001
2002	Ln (January–December)	0.570	0.279	−1.067	2619.403	<0.001
2003	Ln (January–December)	0.645	0.264	−0.996	3601.928	<0.001
2004	Ln (January–December)	0.612	0.239	−0.811	3121.737	<0.001
2005	Ln (January–December)	0.635	0.283	−1.088	3438.869	<0.001
2006	Ln (March–July)	0.721	0.266	−0.811	5112.385	<0.001
2007	Ln (January–December)	0.508	0.236	−0.820	2045.391	<0.001
2008	Ln (January–December)	0.580	0.254	−0.914	2733.455	<0.001
2009	Ln (January–December)	0.614	0.225	−0.719	3142.520	<0.001
2010	Ln (March-August)	0.609	0.266	−0.889	3082.357	<0.001
2011	Ln (March–July)	0.638	0.273	−0.829	3490.340	<0.001
2012	Ln (January–December)	0.638	0.273	−1.028	3494.115	<0.001

Note: Linear regression analysis is made with the model of y = ax + b, where y is NDVImax, x is the optimal cumulative precipitation, which is the logarithmic value of cumulative precipitation in different periods, a is the regression coefficient, b is the constant of the regression equation, R^2^ is the coefficient of determination, F is the F value in the F test and Sig is the statistical significance of the regression model.

**Table 3:** Residual Trend Grade Distribution during 1982–1990, 1982–2000 and 1982–2012.

ID	1982–1990 (%)	1982–2000 (%)	1982–2012 (%)	Land Degradation Degree
D4	1.3	0	0	Extremely severe degradation
D3	8.5	0.3	0.01	Severe degradation
D2	17.1	5.1	1.38	Moderate degradation
D1	25.6	46.8	51.66	Slight degradation
I1	23.5	42.9	44.77	Slight restoration
I2	15.0	4.5	2.05	Moderate restoration
I3	8.3	0.4	0.07	Significant restoration
I4	0.6	0	0.05	Extremely significant restoration

## Appendix A

**Table A1:** RMSE (Root Mean Square Error) Distribution of NDVIpot and Actual NDVI of Northern China.

RMSE	1982–1990 (%)	1982–2000 (%)	1982–2012 (%)
<0.25	81.5%	81.4%	80.8%
0.25–0.5	18.2%	18.2%	18.8%
>0.5	0.3%	0.4%	0.4%
